# Freehand power-assisted pedicle screw placement in scoliotic patients: results on 5522 consecutive pedicle screws

**DOI:** 10.1007/s12306-022-00754-x

**Published:** 2022-08-09

**Authors:** C. Faldini, F. Barile, G. Viroli, M. Manzetti, M. Ialuna, M. Traversari, A. Paolucci, A. Rinaldi, G. D’Antonio, A. Ruffilli

**Affiliations:** IRCCS Istituto Ortopedico Rizzoli, Via Giulio Cesare Pupilli 1, 40136 Bologna, Italy

**Keywords:** Scoliosis, Pedicle screws, Spine, Gertzbein-Robbins scale

## Abstract

Pedicle screws is the current gold standard in spine surgery, achieving a solid tricolumnar fixation which is unreachable by wires and hooks. The freehand technique is the most widely adopted for pedicle screws placing. While freehand technique has been classically performed with manual tools, there has been a recent trend toward the use of power tools. However, placing a pedicle screw remains a technically demanding procedure with significant risk of complications. The aim of this article is to retrospectively evaluate safety and accuracy of free-hand power-assisted pedicle screw placement in a cohort of patients who underwent correction and fusion surgery for scoliosis (both idiopathic and non-idiopathic) in our department. A retrospective review of all patients with scoliosis who underwent surgery and received a postoperative CT scan in our department in a 9-year period was undertaken. Screw density, number and location of pedicle screws were measured using pre and postoperative full-length standing and lateral supine side-bending radiographs. Then, postoperative CT scan was used to assess the accuracy of screw placement according to Gertzbein-Robbins scale. Malpositioned screws were divided according to their displacement direction. Finally, intra and postoperative neurological complications and the need for revision of misplaced screws were recorded. A total of 205 patients were included, with a follow-up of 64.9 ± 38.67 months. All constructs were high density (average density 1.97 ± 0.04), and the average number of fusion levels was 13.72 ± 1.97. A total of 5522 screws were placed: 5308 (96.12%) were grade A, 141 (2.5%) grade B, 73 (1.32%) grade C. Neither grade D nor grade E trajectories were found. The absolute accuracy (grade A) rate was 96.12% (5308/5522) and the effective accuracy (within the safe zone, grade A + B) was 98.6% (5449/5522). Of the 73 misplaced screws (grade C), 59 were lateral (80.80%), 8 anterior (10.95%) and 6 medial (8.22%); 58 were in convexity, while 15 were in concavity (the difference was not statistically significant, *p* = 0.33). Intraoperatively, neither neurological nor vascular complications were recorded. Postoperatively, 4 screws needed revision (0.072% of the total): Power-assisted pedicle screw placing may be a safe an accurate technique in the scoliosis surgery, both of idiopathic and non-idiopathic etiology. Further, and higher quality, research is necessary in order to better assess the results of this relatively emerging technique.

## Introduction

Posterior spinal instrumented fusion using pedicle screws is the current gold standard in the treatment of spinal deformities. In fact, when compared to other constructs (such as hooks and wires), they allow to exert powerful correction forces and to achieve stronger stability [1–4].

However, placing a pedicle screw remains a technically demanding procedure with significant risk of complications: misplaced screws, in fact, can lead to neurologic, vascular, pulmonary and visceral complications [4, 5]. In severe scoliosis, screw placement is particularly challenging due to vertebral bodies rotation and pedicles dysmorphism. These pathological features are mainly present in the apical and periapical regions, on the concave side of the main curve [6, 7]. Pedicle screws placement with freehand technique is currently the most popular choice [3]. Classically, this technique has been performed with manual tools: a gearshift for hole preparation, a manual tap for tapping and a manual screwdriver for insertion. However, while the principles remain the same (perforation, tapping, insertion), there has been a recent trend toward the use of power tools.

The aim of this article is to retrospectively evaluate safety and accuracy of freehand power-assisted pedicle screw placement in a cohort of patients who underwent correction and fusion surgery for scoliosis (both idiopathic and non-idiopathic) in our department.

## Materials and methods

### Study sample

A retrospective review of all patients with scoliosis (both idiopathic and non-idiopathic) who underwent surgery and received a postoperative CT scan in our department between January 2012 and January 2021 was undertaken. Patients with a history of previous spinal surgery and patients who did not receive a postoperative CT scan were excluded.

Follow-up evaluations were performed postoperatively and at last follow-up (minimum 1-year).

Informed consents for participation in the study and for publication of clinical images were obtained from each patient.

### Data collection

Screw density, number and location of pedicle screws were measured using pre and postoperative full-length standing and lateral supine side-bending radiographs. High density was defined above 1.61 screws per level [8].

Then, postoperative CT scan was used to assess the accuracy of screw placement according to Gertzbein–Robbins scale (from grade A to E: A, perfect intra-pedicular localization; B, 0–2 mm misplacement; C, 2–4 mm; D 4–6 mm; E > 6 mm misplacement) [9]. An “in–out-in” screw placement was not graded differently.

The slice with the largest deviation from the pedicle was chosen for grading. As per radiological and clinical convention, a pedicle breach up to 2 mm is considered to be within the safe zone for neural structures: hence, grades A and B were considered to be acceptable [10].

Moreover, malpositioned screws were divided according to their displacement direction (anterior, medial, lateral) and their position in convexity or concavity of the curve. All measures were taken independently by two experienced spine surgeons (FB and GV) who did not participate to surgeries: when in doubt, the worst grading was considered.

Finally, intra and postoperative neurological complications and the need for revision of misplaced screws were recorded.

All screws were placed with power-assisted freehand technique, as described by Faldini et al. [11].

### Statistical Analysis

We analyzed malposition rates and basic descriptive statistics using Chi-Square and Fisher exact tests. Spearman Rho’s was used to make correlations. A *p* value < 0.05 was considered statistically significant. All statistical analyses were performed with IBM SPSS v26.0 (SPSS Inc., Chicago, Illinois).

## Results

Two hundred five patients were included, with a follow-up of 64.9 ± 38.67 months. The mean age at surgery was 28.2 ± 18.65 years (range 12–72). All constructs were high density (average density 1.97 ± 0.04), and the average number of fusion levels was 13.72 ± 1.97. (Table 1).

**Table 1:** Clinical characteristics of the cohort

	Mean	Standard deviation
Age	28.2	18.6
Fused levels	13.7	1.9
Screw density	1.9	0.1
Follow-up	64.9	38.6

A total of 5522 screws were placed: 5308 (96.12%) were grade A, 141 (2.5%) grade B, 73 (1.32%) grade C. Neither grade D nor grade E trajectories were found. Therefore, the absolute accuracy (grade A) rate was 96.12% (5308/5522) and the effective accuracy (within the safe zone, grade A + B) was 98.6% (5449/5522) (Table 2).

**Table 2:** Misplaced Screws

	Total: 5522 screws			
	Medial	Lateral	Anterior	Total
Grade B	29	98	14	141 (2.5%)
Convex-side	16	75	6	97
Concave-side	13	23	8	44
Grade C	6	59	8	73 (1.32%)
Convex-side	4	48	6	58
Concave-side	2	11	2	15

Of the 73 misplaced screws (grade C), 59 were lateral (80.80%), 8 anterior (10.95%) and 6 medial (8.22%); 58 were in convexity, while 15 were in concavity (the difference was not statistically significant, *p* = 0.33).

Intraoperatively, neither neurological nor vascular complications were recorded. Postoperatively, 4 screws needed revision (0.072% of the total): in 3 cases, 1 grade C lumbar screw (with medial breach) was removed (L2 with medial breach in 1 case, L5 with lateral breach in 1 case and L5 with anterior breach close to iliac artery in 1 case); in 1 patient, 1 screw was removed for a slight (grade B) breach of the anterior wall of T3, with the screw tip that appeared close to the esophagus.

## Discussion

Since Roy-Camille first introduced pedicle screw fixation of the spine, it has become the most popular form of posterior spinal instrumentation when correcting spinal deformities [13]. However, safety and reliability are still a major concern, considering the possible catastrophic consequences that a misplaced screw may have. The presented study reports the results, in terms of safety and accuracy, of freehand power-assisted pedicle screw placing in a cohort of 205 scoliotic patients (both idiopathic and non-idiopathic). The technique proved to be precise and safe, allowing to place 98.6% of the screws within the safe zone, while keeping the screw revision rate as low as 0.072%. These results are on the lower spectrum of the reported perforations rate in the literature regarding pedicle screw instrumentation in scoliosis [5].

Among spinal surgeons, there has been a recent trend toward the use of power for pedicle tract preparation and screws placement [14–16]. In fact, there are some advantages that make power appealing compared to the manual technique: shorter screw insertion phase [16], shorter fluoroscopy time [14], reduced wobble phenomenon [17], lower rates of screw failure [14] and of revision per screw [15]. Moreover, power-assisted technique may protect against the risk of overuse injuries, allowing to keep the average muscle exertion of the surgeon under a safe threshold up to 100% of a procedure time [18]. This may be crucial in order to maximize productivity and longevity of a surgeon’s practice, since a survey of members of the Scoliosis Research Society reveals rates of neck pain, rotator cuff disease, lateral elbow epicondylitis, and cervical radiculopathy at 3 X, 5 X, 10 X, and 100 X greater than that of the general population [19].

Some technical aspects must then be considered. First, the slowly rotating drill bit almost acts like a flexible ball-tipped probe offering an optimal tactile feedback that allows the surgeon to feel if the threads are cutting into the soft cancellous bone of the pedicle channel versus into the hard cortical bone of the pedicle wall [11]. Finally, if the tract trajectory is unsatisfactory, a new tract can be created without any significant compromise of the pedicle anatomy. Conversely, the manual probe, due to its larger diameter and its less accurate advancing, tends to be less forgiving when creating multiple tracts, resulting in the confluence of the various tracts and ultimately leading in a decreased bone purchase of the screw [11]. When comparing our results with the other power-assisted cohorts published in the literature, only Yan et al. [16] routinely evaluated the accuracy of pedicle screws with CT scan, showing a 89% of thoracic screws within the safe zone. While this accuracy rate is lower than the 98.6% rate reported in the presented study, it must be acknowledged that Yan et al. only considered thoracic pedicle screws in the analysis, which are at greater risk of perforation because of both intrinsic (smaller dimensions) and pathologic (pedicle dysplasia in scoliotic patients) pedicle anatomy in this region. The 0.072% revision per screw rate in our cohort is line with the reported rate in other power-assisted studies, ranging from 0 to 0.14% [14–16].

Despite the recent introduction of multiple aids in pedicle screws placement, such as 2D and 3D fluoroscopic navigation, CT navigation, robot-assisted placement and patient-specific 3-D printed guides [ref], freehand placement still remains the most widely used technique. In fact, all these systems may have cost-related issues as well as limited availability at some institutions or countries. Moreover, each system has some specific drawbacks that must be acknowledged: 2D fluoroscopic navigation showed lower pedicle screw placement accuracy compared to 3D fluoroscopic and CT navigation [20] systems, which are in turn associated with significant radiation exposure [21, 22]; robot-assisted placement requires prolonged operative times [23] and may have some malpositioning issues because of skiving [24]; patient-specific guides may lead to excessive ligamentous dissection in order to maximize guide-bone contact areas. In light of that it must be stated that all of these technologies are still valid alternatives to freehand technique, especially in specific situations. For example, revisions of residual deformity cases, in which a prior posterior spinal fusion has been performed: in this setting, posterior anatomical landmarks are lost, making freehand technique unsafe at times. Moreover, these cases often require complex tricolumnar osteotomies, so, an aid in screw placement may allow the surgeon to save focus and energies for the next, more critical, surgical steps.

Finally, this study does not come without important limitations: its retrospective design, the absence of a comparison group and the relatively limited number of patients may underpower the presented results. However, this study may increase the level of evidence regarding power-assisted pedicle screw placing since it is the only study in the literature, along with the one by Yan et al. [16], that objectively evaluated the accuracy rate with a post-op CT scan.

## Conclusion

Power-assisted pedicle screw placing may be a safe an accurate technique in the scoliosis surgery, both of idiopathic and non-idiopathic etiology. Further, and higher quality, research is necessary in order to better assess the results of this relatively emerging technique.

## Data Availability

The data that support the findings of this study are available from the corresponding author upon reasonable request.
